# Deletion of kinin receptor B2 enhances orthodontic tooth movement and alveolar bone remodeling

**DOI:** 10.1371/journal.pone.0318436

**Published:** 2025-02-28

**Authors:** Natália Couto Figueiredo, Mitchell Piacsek, Carina Cristina Montalvany-Antonucci, Mariana de Souza Santos, Flávio Almeida Amaral, Mauro Martins Teixeira, Tarcília Aparecida Silva, Soraia Macari, Subramanya Pandruvada, Ildeu Andrade Jr

**Affiliations:** 1 Graduate Program in Dentistry, School of Dentistry, Pontifical Catholic University of Minas Gerais, Belo Horizonte, Minas Gerais, Brazil; 2 Department of Biomedical and Community Health Sciences, Division of Basic Science Research, College of Dental Medicine, Medical University of South Carolina, Charleston, South Carolina, United States of America; 3 Department of Restorative Dentistry, School of Dentistry, Federal University of Minas Gerais, Belo Horizonte, Minas Gerais, Brazil; 4 Department of Physiology and Biophysics, Institute of Biological Sciences, Federal University of Minas Gerais, Belo Horizonte, Minas Gerais, Brazil; 5 Department of Biochemistry and Immunology, Biological Science Institute, Federal University of Minas Gerais, Belo Horizonte, Minas Gerais, Brazil; 6 Department of Oral Surgery, Pathology, and Clinical Dentistry, School of Dentistry, Federal University of Minas Gerais, Belo Horizonte, Minas Gerais, Brazil; 7 Department of Orthodontics and Dentofacial Orthopedics, School of Dental Medicine, University of Pittsburgh, Pittsburgh, Pennsylvania, United States of America; Max Delbruck Centrum fur Molekulare Medizin Berlin Buch, GERMANY

## Abstract

The kallikrein-kinin system (KKS) is a complex enzymatic system involved in multiple biological processes, particularly inflammation. The system’s peptides exert broad effects through two receptors, B1 (B1R) and B2 (B2R), expressed in various cell types, including osteoblasts. However, the impact of this system on bone remodeling induced by mechanical force needs to be better understood. This study aimed to elucidate the role of the B2 kinin receptor in bone phenotype and remodeling under mechanical stress. Orthodontic forces were applied to the upper first molars of B2R^−/−^ mice and wild-type controls. Bone parameters, bone cellular counts, expression of inflammatory biomarkers, and osteoblast and osteoclast differentiation and activity were assessed using microtomography, histological analysis, real-time polymerase chain reaction (qPCR), and in vitro bone cell cultures, respectively. The results revealed that B2 receptor deficiency significantly altered maxillary bone architecture, reduced trabecular thickness, increased orthodontic tooth movement, and spontaneous alveolar bone loss (ABL). Histological analysis showed a higher number of osteoclasts in B2R^−/−^ mice, with no significant change in osteoblast counts. Molecular analysis indicated elevated levels of RANK, RANKL, OPG, RANKL/OPG, IL-1β, and B1 receptor expression in B2R^−/−^ mice, while in vitro studies confirmed enhanced osteoclast numbers and activity in B2R^−/−^ cells. In conclusion, this study underscores the critical roles of kinin receptors in regulating alveolar bone remodeling, with B2R deletion leading to increased osteoclastic activity and bone loss. The compensatory upregulation of B1Rs in the absence of B2Rs suggests functional redundancy. However, the B2R^−/−^ phenotype emphasizes the complex involvement of the KKS pathway in bone physiology, suggesting avenues for further research into bone pathophysiology and potential therapeutic approaches.

## Introduction

The alveolar bone, a crucial component of the tooth-supporting structure, undergoes continuous physiological remodeling to preserve its structural integrity and functional capacity [1]. This dynamic process involves coordinated osteoclast-mediated bone resorption and osteoblast-mediated bone formation [2,3]. However, within the oral environment, inflammatory events caused by biological or mechanical stimuli can disrupt this balance, affecting the morphological and biomechanical bone properties [4,5]. Several mediators participate in this process, such as cytokines, chemokines, growth factors, and hormones [6]. Notably, the kallikrein-kinin system (KKS) has been shown to play a pivotal role in modulating these events, highlighting its importance in bone biology and inflammation [7].

The KKS is an endogenous cascade involved in several pathophysiological phenomena, including inflammation, vasodilation, increased vascular permeability, and pain [8,9]. The system comprises the precursors of high and low molecular-weight kininogens, which are cleaved by tissue- or plasma-kallikreins, producing four active peptides: Bradykinin (BK), Lys-bradykinin (Lys-BK) and their des-Arg^9^ metabolites, des-Arg^9^-BK and lys-Des-Arg^9^-BK, respectively [10]. These kinin peptides exert their effects by interacting with two G-protein-coupled receptors (GPCRs): the B2 receptor (B2R) and the B1 receptor (B1R). The B2R is constitutively expressed in various cell types and exhibits a higher affinity for BK and Lys-BK. In contrast, the B1R is typically absent or expressed at very low levels under physiological conditions but is rapidly upregulated in response to inflammatory stimuli [8]. The B1R exhibits greater affinity for the des-Arg metabolites, des-Arg_9_-BK, and Lys-des-Arg_9_-BK, which are generated from BK and Lys-BK by the enzymatic action of carboxypeptidases. Unlike BK and Lys-BK, which are rapidly degraded by angiotensin-converting enzyme (ACE), des-Arg metabolites are resistant to ACE cleavage and play a key role in sustaining inflammatory responses through B1R activation [11,12].

The kinin signaling pathway is known for its potential to stimulate bone resorption and synergistically enhance the bone-resorbing effects triggered by various mechanisms [10]. Osteoblasts express B1R and B2R, which can interact with IL-1 and TNF-α to cause a synergistic increase in prostaglandin production [13,14]. This interaction between kinins and cytokines also leads to enhanced COX-2 mRNA expression and is associated with the upregulation of receptor activator of NF-κB ligand (RANKL) mRNA and protein levels [15]. Since RANKL is essential for osteoclast differentiation and activation, this finding likely explains the synergistic effect on bone resorption [15,16]. Interestingly, it has been reported that mice lacking both subtypes of kinin receptors had bone mineral loss [17].

However, the specific roles of kinin receptors in bone remodeling induced by mechanical stimuli still need to be fully understood. Therefore, this study aimed to assess the effects of ubiquitously expressed B2R deletion on physiological and mechanically induced bone remodeling using a well-established mouse model of orthodontic tooth movement (OTM) [18,19]. The findings of this investigation, which focused on whether kinin receptor deficiency influences maxillary bone remodeling and the local production of inflammatory molecules in mice, have potential implications for developing novel therapeutic strategies for bone-related diseases. Additionally, the possible association between the B2 receptor-deficient (B2R^−/−^) phenotype and the differentiation of bone cells in vitro was assessed, providing further insights into the role of KKS in bone remodeling.

## Materials and methods

### Experimental animals

Eight- to ten‐week‐old healthy male C57BL6/J wild-type (WT) mice, along with B2R^−/−^ mice [20], were included in this study. The sample size calculation was based on a previous study from our group [18]. These animals were sourced from the Federal University of Minas Gerais Animal House. They were maintained under standard conditions with a 12-hour light/dark cycle, controlled temperature (24°C ± 2°C), and free access to commercial chow and drinking water. Throughout the experimental period, the location of animal cages was rotated regularly to prevent location-based bias. Mice were monitored daily for signs of severe pain, distress, or significant weight loss, and no animals reached the humane endpoints. This study followed a pre-defined protocol approved by the Ethics Committee in Animal Experimentation of the Federal University of Minas Gerais (protocol 394/2015), and followed the ARRIVE guidelines established by the National Centre for the Replacement, Refinement, and Reduction of Animals in Research [21]. Randomization was not applicable as their genetic background inherently defined their allocation.

### Experimental protocol for bone remodeling induced by OTM

Alveolar bone remodeling was induced by OTM, as previously described [18,19]. Briefly, the male mice were anesthetized with 0,2 ml of xylazine (0,02 mg/ml) and ketamine (50 mg/ml) solution. A nickel-titanium 0.25 x 0.76 mm coil spring (Lancer Orthodontics, San Marcos, CA) was bonded using light-cured resin (Transbond; 3M Unitek, Monrovia, CA) between the maxillary right first molar and maxillary incisors. The force of 0.35 N was applied and calibrated with a tension gauge (Shimpo Instruments, Itasca, IL), with no reactivation during the experiment. The left maxilla was the control. No adverse events were observed during the experimental period.

### Microtomography (MicroCT) imaging

For the MicroCT analysis, mice were euthanized after 12 days of mechanical loading (n =  5 per group). Maxillary alveolar bones were fixed in 10% neutral buffered formalin for 48 hours and scanned using a MicroCT system (Skyscan 1172 X-Ray microtomograph; Skyscan, Aartselaar, Belgium). The calibration was carried out with known-density calcium hydroxyapatite phantoms (Skyscan). High-resolution scans with an isotropic voxel size of 18 μm were acquired (50 kv, 0.5 mm aluminum filter, 0.5° rotation angle). Contouring methods were used to delineate the region of interest to be analyzed as described [22].

Trabecular morphometry was measured within the furcation area of the first molar root at the control side. The parameters measured included bone mineral density (BMD, g/cm^−3^), percent bone volume/total volume (BV/TV, %), trabecular thickness (Tb.Th, μm), trabecular number (Tb.N, μm^−1^) and trabecular separation (Tb.Sp, μm). Alveolar bone loss (ABL) was measured on the control side by determining the area (μm²) between the cemento-enamel junction (CEJ) and the alveolar bone crest (ABC) of the first, second, and third maxillary molars. Measurements were performed using Fiji software (National Institutes of Health, USA), where the CEJ and ABC were manually identified, and the region of interest was delineated to calculate the area [22]. The amount of OTM (μm) was measured by comparing the shortest linear distance between the CEJ of the first and second maxillary molars on the right (experimental) and left (control) sides of the same animal using the linear measurement tool of CTAn software (Bruker-MicroCT) [23].

### Histological analysis

Maxillae were carefully dissected, fixed in 10% formaldehyde buffered solution for 48 h, and decalcified with 14% ethylenediaminetetraacetic acid (EDTA) (pH 7.4) for 21 days. The anterior fragment containing the incisors was removed, and the right and left hemimaxillae were isolated and then embedded in paraffin [18]. The blocks were sectioned sagittally at a thickness of 5 μm. The samples were stained with tartrate-resistant acid phosphatase (TRAP) (Sigma-Aldrich) and counterstained with hematoxylin or Masson’s trichrome for histopathological examinations.

Osteoclasts, identified as TRAP-positive multinucleated cells on the mesial side of the distal-buccal root of the first molar, and osteoblasts, identified by Masson’s trichrome staining on the distal side of the same root, were counted. Evaluations were conducted in three histological sections per animal at X40 magnification (n =  5 per group).

### Quantitative real-time PCR (qPCR)

Mice were euthanized after 72 hours of OTM for molecular analysis (n =  5 per group). Maxilla samples, excluding gingival tissue and oral mucosa, were frozen in liquid nitrogen and stored at −80°C. Total RNA was extracted from the periodontal ligament of the upper molars and the surrounding alveolar bone from both the control and OTM sides using an RNeasy kit (Qiagen Inc, Valencia, CA), following the manufacturer’s instructions. Following RNA extraction, complementary DNA (cDNA) was synthesized from 2,5 μg of RNA via reverse transcription reaction using the SuperScript VILO Master Mix reverse transcriptase enzyme (Thermo Fisher Scientific, MA, USA). The target genes analyzed included receptor activator of nuclear factor‐kappa B (RANK), RANKL, osteoprotegerin (OPG), BDKRB1, interleukin‐1β (IL-1β), interleukin‐6 (IL-6) and tumor necrosis factor-alpha (TNF-α). mRNA levels were quantified using a Rotor-Gene Q system (QIAGEN, Hilden, Germany) with the SYBR^®^ Green PCR Master Mix kit (Thermo Fisher Scientific). The PCR conditions were initial denaturation at 95°C for 10 minutes, followed by 40 cycles of 95°C for 15 seconds, and annealing at 60°C for 1 minute. The relative levels of gene expression were calculated using the cycle threshold (Ct) method and the 2 −ΔΔCt formula, with glyceraldehyde 3-phosphate dehydrogenase (GAPDH) as the internal control. The sequences of all primers used in qPCR analysis and the expected product sizes are summarized in Table 1.

**Table 1:** Primers used for qPCR analyses.

Target gene	Primer sequences	Product size (bp)
RANKL	Fwd CGTGCAGAAGGAACTGCAACAC Rev TGGTGAGGTGTGCAAATGGCT	131
OPG	Fwd TCATCCAAGACATTGACCTCTGTGA Rev GCTGCTCGCTCGATTTGCAG	165
RANK	Fwd AGCATCCCTTGCAGCTCAACA Rev TTCCGTTGTCCCCTGGTGTG	149
BDKRB1	Fwd CCATAGCAGAAATCTACCTGGCTAAC Rev GCCAGTTGAAACGGTTCC	102
IL-1β	Fwd TGCCACCTTTTGACAGTGATGA Rev ATCAGGACAGCCCAGGTCAA	101
TNF-α	Fwd GCGACGTGGAACTGGCAGAA Rev TTTGGGAACTTCTCATCCCTTTGGG	175
IL-6	Fwd TTCCATCCAGTTGCCTTC Rev TTGGGAGTGGTATCCTCTGTGA	101
GAPDH	Fwd GGCAAATTCAACGGCACAGT Rev AGATGGTGATGGGCTTCCC	70

Fwd, forward primer; Rev, reverse primer; bp, size of the amplified product in base pairs.

### Osteoclast differentiation and resorption assays

Primary bone marrow cells (BMCs) were isolated from the femurs and tibiae of WT and B2R^−/−^ mice (n =  3 per group). Cells were cultured in complete minimum essential medium alpha (α MEM, with no nucleosides) (Thermo Fisher Scientific, Waltham, MA, USA) supplemented with 10% fetal bovine serum (Gibco; Thermo Fisher Scientific), 100 U/mL penicillin and streptomycin, and M‐CSF (30 ng/mL, R&D Systems Europe). The cultures were maintained at 37°C in a 5% CO_2_ humidified atmosphere for three days to induce pre-osteoclast formation. Subsequently, adherent cells were collected and seeded at 2.5 ×  10^4^ cells per well in 96-well plates. Stimulation with M-CSF (30 ng/mL, R&D Systems Europe) and RANKL (50 ng/mL, R&D Systems) was performed, and cultures were continued for seven days [24]. Endpoint analyses included formalin fixation of cultures and staining for tartrate‐resistant acid phosphatase (TRAP) activity (Sigma‐Aldrich, Saint Louis, MO, USA), following the manufacturer’s instructions. Osteoclasts were identified as TRAP + cells containing more than three nuclei.

Additionally, independent cell viability assessments were performed using the MTT (3-(4,5-dimethylthiazol-2-yl)-2,5-diphenyltetrazolium bromide) assay for all experimental groups. For the MTT assay, 10 μL of the MTT (5 mg/mL) was added to each well containing 90 μL of medium. The plates were incubated at 37°C for 4 hours. After incubation, 50 μL of dimethyl sulfoxide (DMSO) was added to each well, and the plates were agitated to dissolve the dark blue formazan crystals. Absorbance was measured at 540 nm using a spectrophotometer [25].

Osteoclast activity was evaluated by seeding BMCs onto an osteoassay surface 96-well plate (Corning, NY, USA) to measure resorption-pit formation, as described previously [23]. After ten days of culture, cells were removed from the bone surface, and the resorbed bone area was quantified from acquired images of pits using ImageJ software.

### Osteoblast cultures and alizarin red staining

Primary BMCs were isolated from the femurs and tibiae of WT and B2R^−/−^ mice (n =  3 per group). BMCs were cultured in minimum essential medium alpha (α MEM, with nucleosides) (Thermo Fisher Scientific, Waltham, MA, USA) supplemented with 10% fetal bovine serum (Gibco; Thermo Fisher Scientific), 100 U/mL penicillin and streptomycin. The osteogenic medium is prepared supplementing with 10% FBS, 10 mM β-glycerophosphate, 0.1 μM dexamethasone, 0.5 mM ascorbic acid, 100 U/mL penicillin and streptomycin. The osteogenesis process started when the BMCs reached 80% confluency by replacing cell culture media with osteogenic medium, which was changed twice a week. Alizarin red staining (ARS) was used to detect and quantify calcium within the deposited mineral matrix. The cells were cultured on the four replicates for 28 days for this assay. Cells were fixed with 10% buffered formalin for 20 minutes at room temperature, washed with deionized water, and stained with 40 mM ARS solution (pH 4.2) for 20 minutes. After removing the staining solution, the cells were washed with deionized water three times and photographed to capture representative images. Quantification of ARS was performed using an acetic acid extraction method [25]. Cells were incubated with 10% acetic acid for 30 minutes at room temperature under agitation, and the lysates were neutralized with ammonium hydroxide. The final solution was loaded into a 96-well plate in triplicate, and absorbance was measured at 405 nm [25].

Data collection and analysis for all described experiments were performed by researchers who were blinded to the group assignments.

### Statistical analysis

The data were expressed as the mean ±  standard deviation (SD). Statistical analyses were performed using the Student’s T-test (two-tailed) to compare the means between groups. The results obtained from all evaluations were processed with GraphPad Prism version 5.01 (GraphPad Software, San Diego, CA). The level of significance for all statistical tests was predetermined at 5%. All data supporting the findings of this study are available in the supporting information (S2 Dataset).

## Results

### Lack of B2R favors OTM, ABL and deteriorates maxillary microarchitecture

**Fig 1:**
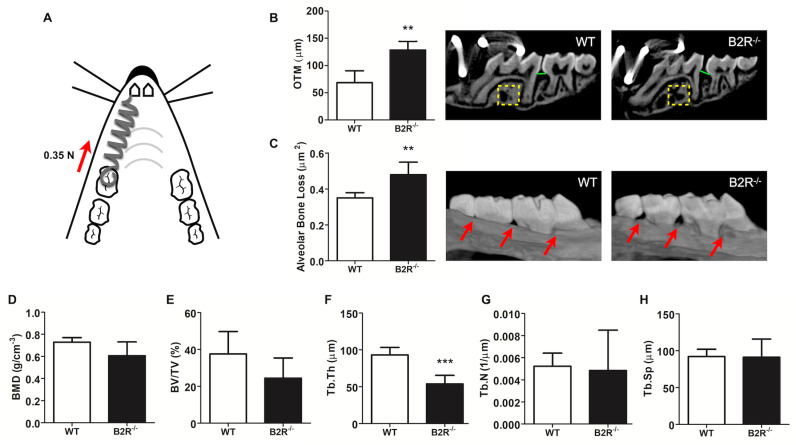
MicroCT parameters illustrate the effects of B2 kinin receptor deletion on alveolar bone (control side) and OTM. (A) Representative image of the OTM model. (B) Quantification of OTM with corresponding representative images. OTM was determined by calculating the difference in the shortest linear distance between the CEJ of the first and second maxillary molars on the experimental (right) and control (left) sides of the same animal, using a split-mouth design. Yellow dotted squares denote the analyzed area for microarchitectural parameters (control side), and green lines indicate the distance between the first and second molars, highlighting the extent of tooth movement. (C) ABL area (control side). The ABL was determined by calculating the area between the CEJ and the ABC of the first, second, and third molars on the control side. Red arrows indicate the levels of the ABC. (D) Bone Mineral Density (BMD, g/cm^−3^). (E) Bone Volume/Total Volume (BV/TV, %). (F) Trabecular Thickness (Tb.Th, μm). (G) Trabecular Number (Tb.N, μm^−1^). (H) Trabecular Separation (Tb.Sp, μm). ** Significant difference from control (*p* < 0.01). *** Significant difference from control (*p* < 0.001). Data are expressed as the mean ±  SD. Statistical analysis was performed using Student’s *t-*test (n =  5 animals per group, totaling 10 animals).

MicroCT analysis showed a disruption of maxillary bone architecture, as demonstrated by the alteration of some bone parameters (Fig 1A–H). B2R^−/−^ mice exhibited reduced trabecular thickness and increased OTM after 12 days (Fig 1B, F), and showed a spontaneous ABL, marked by increased CEJ-ABC area, compared with WT animals (Fig 1C). No statistical differences were found in BMD, BV/TV, Tb.N, and Tb.Sp between the experimental and control groups (Fig 1D, E, G, H).

### B2R^−/−^ mice showed increased numbers of osteoclasts in alveolar bone

**Fig 2:**
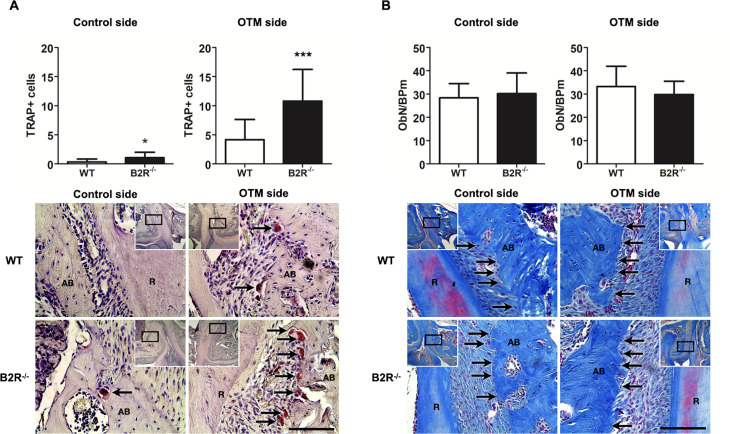
Bone cell counts in the tooth surrounding bone. (A) Quantification and corresponding representative images of TRAP-positive osteoclasts counted on the maxillary bone in WT and B2R^−/−^ groups on the control side and under OTM. Black arrows indicate osteoclasts. (B) Quantification and corresponding representative images of the bone lining osteoblasts counted on the maxillary bone of WT and B2R^−/−^ mice on the control and OTM side. Black arrows indicate osteoblasts. AB, alveolar bone; R, root; ObN/BPm, osteoblast number/bone perimeter. Scale bar =  100 μm. *  Significant difference from control (*p* < 0.05). *** Significant difference from control (*p* < 0.001). Data are expressed as the mean ±  SD. Statistical analysis was performed using Student’s *t-*test (n == 5 animals per group, totaling 10 animals).

Histological analysis revealed a significant increase in osteoclast numbers in the alveolar bone of B2R^−/−^ mice (Fig 2A). Specifically, B2R^−/−^ mice had more TRAP-positive osteoclasts on the mesial side of the bone adjacent to the distal-buccal root of the maxillary first molar, on both the control and experimental sides, compared to WT mice. There were no significant differences in osteoblast numbers among the groups (Fig 2B).

### Alveolar bone of B2R ^−/−^ mice exhibited changes in the expression of specific biomarkers

**Fig 3:**
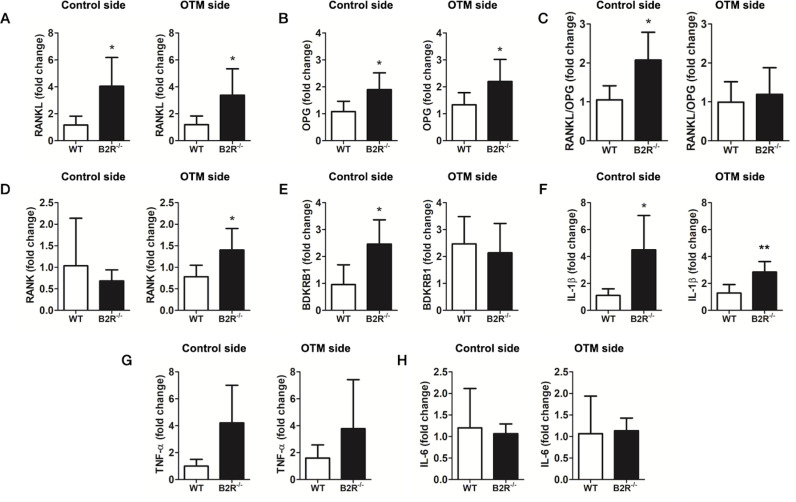
Gene expression of biomarkers in alveolar bone, at control and OTM sides. (A) RANKL. (B) OPG. (C) RANKL/OPG ratio. (D) RANK. (E) BDKRB1. (F) IL-1β. (G) TNF-α. (H) IL-6. *  Significant difference from control (*p* < 0.05). ** Significant difference from control (*p* < 0.01). Data are expressed as mean ±  SD. Statistical analysis was performed using Student’s *t-*test (n =  5 animals per group, totaling 10 animals).

To further explore how kinins can regulate bone remodeling in the maxilla, we investigated whether the B2R deletion could interfere with the mRNA expression of B1R and key biomarkers involved in the bone inflammatory process (Fig 3A–H). In parallel to the altered bone phenotype, spontaneous ABL, and increased osteoclast number in the maxillary alveolar bone of B2R^−/−^ mice, molecular analysis showed an increase in RANKL, OPG, and IL-1β expression on both control and experimental sides of these animals (Fig 3A, B, F). The RANKL/OPG ratio was higher in the control side of the B2R^−/−^ group compared to WT (Fig 3C). RANK expression was significantly increased on the experimental side of B2R^−/−^ animals, correlating with greater OTM (Fig 3D). Additionally, B1R expression was significantly higher on the control side of B2R^−/−^ mice than WT (Fig 3E). Expression levels of pro-inflammatory cytokines IL-6 and TNF-α were similar across all groups, irrespective of mechanical loading (Fig 3G, H).

### Inhibition of B2R regulates the differentiation and activity of bone cells *in vitro*

**Fig 4:**
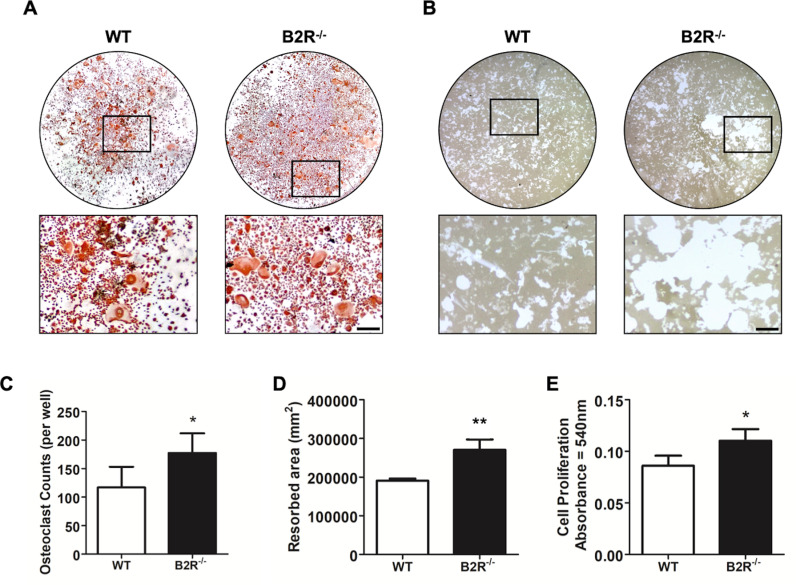
Effects of B2R deletion on osteoclast differentiation and activity *in vitro.* (A) Representative image of bone marrow cells differentiated into osteoclasts from WT and B2R^−/−^ mice (scale bar =  200 μm). (B) Representative image of resorption pit areas. (C) Count of TRAP-positive osteoclasts from WT and B2R^−/−^ mice. (D) Quantification of resorption areas by osteoclasts derived from WT and B2R^−/−^ mice. (E) MTT assay to assess the viability of cells after seven days of differentiation. *  Significant difference from control (*p* < 0.05). ** Significant difference from control (*p* < 0.01). Data are expressed as the mean ±  SD. Statistical analysis was performed using Student’s *t-*test (n =  3 animals per group, totaling six animals).

**Fig 5:**
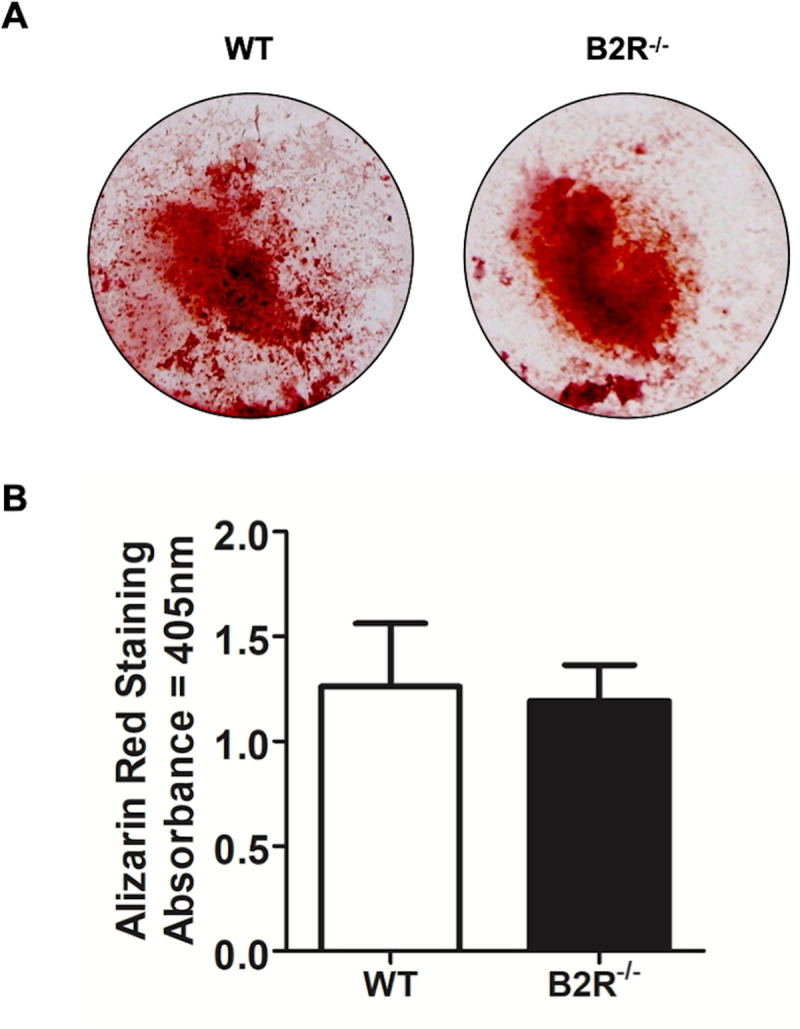
B2R deletion does not impact the differentiation of osteoblasts *in vitro.* (A) Representative image of osteoblast bone marrow cell differentiation of WT and B2R^−/−^ mice. (B) Quantification of areas of mineralization expressed in optical density. Data are expressed as the mean ±  SD. Statistical analysis was performed using Student’s *t-*test (n =  3 animals per group, totaling six animals).

To determine whether the bone phenotype observed in B2R^−/−^ mice is related to altered bone cells’ differentiation and activity, the study assessed the in vitro culture of BMCs isolated from these knockout mice compared to WT controls (Fig 4A–E). By day 7 of differentiation, there was a noticeable increase in the number of TRAP-positive osteoclasts in the B2R^−/−^ group compared to WT cells (Fig 4A, C). Additionally, B2R^−/−^ osteoclasts exhibited larger resorption pit areas and higher cell proliferation, as measured by MTT, compared to the WT group (Fig 4B, D, E). There was no difference in osteoblast differentiation or activity, as indicated by the similar formation of mineralized nodules (Fig 5).

## Discussion

This study uncovers the role of kinin receptors in alveolar bone remodeling induced by mechanical strain. Previous studies have demonstrated that the KKS pathway is a complex system capable of exhibiting both pro- or anti-inflammatory effects, with the influence of kinins and their receptors varying based on disease, target organs, and pathological conditions [26]. The findings, indicating that B2R deletion negatively impacted alveolar bone, have significant implications for bone biology and related fields. Importantly, these findings also hold potential promise for clinical applications, offering hope for improved treatments in the future.

This receptor regulates the physiological bone remodeling process and the remodeling induced by mechanical force. Notably, B2R deletion leads to increased spontaneous ABL, deterioration of bone microarchitecture, and augmented OTM. These outcomes are primarily associated with elevated bone resorption, as evidenced by a higher number of osteoclastic cells in situ, increased expression of proinflammatory mediators and osteoclastogenesis-related mediators, and a greater potential for enhanced osteoclastic cell proliferation and activity in vitro.

Through MicroCT analysis, this study demonstrated a significant reduction in trabecular thickness, spontaneous ABL, and greater OTM in B2R^−/−^ mice. These findings align with and reinforce previous research, which reported significant reductions in bone mineral density in mice lacking kinin receptors [17,27,28]. The absence of the B2R has systemic effects in multiple tissues, extending beyond bone remodeling [27]. This condition was associated with increased oxidative stress, leading to mitochondrial damage, readily detectable in metabolically active cells and can induce apoptosis in some actively dividing cells. Moreover, the B2R partially exerts its effects through the downstream formation of nitric oxide (NO), which regulates cardiac oxygen consumption, a mechanism absent in B2R^−/−^ mice [29]. Additionally, a decrease in intracellular NO due to the absence of the BK B2R [30] may impair aerobic metabolism, increasing oxidative stress.

Further supporting this, it has been shown that the absence of B2R leads to a generalized enhancement of senescence-associated phenotypes across multiple tissues, including alopecia, osteoporosis, kyphosis, and testicular atrophy. These phenotypes are accompanied by increased markers of oxidative stress, such as elevated 8-hydroxy-2′-deoxyguanosine (8-OHdG), and upregulation of genes related to senescence and apoptosis, including TGF-β1, p53, and FOXO1. Together, these changes culminate in cellular apoptosis and tissue degeneration, highlighting the systemic role of B2R in maintaining cellular and mitochondrial integrity [27].

It was previously described that B2R expression is also up-regulated against inflammation [8]. However, this receptor undergoes rapid desensitization and internalization after binding to its respective effector peptides. In contrast, the activated B1R resists desensitization and internalization with sustained and prolonged activity [11,12]. Consistent with these findings, our study also demonstrated that osteoclasts derived from B2R^−/−^ animals exhibited significant resorptive activity, which may have contributed to the deleterious effects observed in alveolar bone. These findings significantly contribute to our understanding of the role of kinin receptors in bone health and disease. Further research is needed to explore how the absence of kinin receptors affects other critical bone biomodulators.

The KKS components, such as ACE, carboxypeptidase M (CPM), and tissue kallikrein, may also play significant roles in bone metabolism and cell function. ACE, known for its role in modulating BK levels, has been associated with enhanced bone health. Specifically, ACE inhibition has been shown to improve bone mineral density and strength, suggesting that ACE, through its effects on BK, may influence bone metabolism [31]. However, there is limited direct evidence regarding the involvement of CPM and tissue kallikrein in bone cell function. CPM plays a role in kinin metabolism, particularly by generating des-Arg BK metabolites that bind to the B1R. While these metabolites are known to contribute to inflammatory responses, their direct effects on bone cells remain less explored [8]. Tissue kallikrein, which is involved in activating kininogens, has similar implications in inflammation but has yet to be thoroughly studied in the context of bone cells [8,32].

Given the evidence supporting the role of the KKS in inflammation and bone resorption, these components may provide valuable insights into bone remodeling. For instance, pharmacological inactivation of the B2R and B1R has shown promise in controlling inflammation and modulating bone resorption in pathological conditions such as arthritis, periodontitis, and osteomyelitis [33]. This approach suggests potential for localized therapeutic applications, targeting receptor activity in specific tissues to mitigate deleterious effects while preserving physiological functions. Further studies focusing on localized receptor inhibition could yield critical insights into the translational potential of these strategies [8,33].

Osteoclastogenesis and bone resorption activity are regulated by numerous inflammatory mediators, particularly the RANK/RANKL/OPG system [3,34]. The effects on alveolar bone in B2R^−/−^ mice prompted an evaluation of inflammatory regulators in their periodontium compared to the WT group. Molecular analysis revealed that the deletion of B2R led to a significant increase in the expression of IL-1 RANK, RANKL, and OPG, with the increase in RANKL being proportionally more substantial than that of OPG, resulting in a raised RANKL/OPG ratio. The significant enhancement of B1R expression, a crucial modulator of T cells, by stimulating RANKL production via IL-17 [28] may explain the higher RANKL/OPG ratio and increased osteoclast numbers in the alveolar bone of B2R^−/−^ mice. Interestingly, despite the up-regulation of the RANKL/OPG ratio in the absence of B2R under regular conditions, the mechanical force application induced a notable increase in RANK expression, likely contributing to the higher number of TRAP-positive cells and greater OTM. Moreover, the naturally impaired bone phenotype is characterized by reduced Tb.Th and spontaneous ABL may have further amplified OTM.

Increased B1R expression in the B2R^−/−^ group may reflect a compensatory change, as B1R is markedly upregulated in various tissues of B2R^−/−^ mice, indicating functional redundancy between the receptors [35,36]. The literature also highlights that the B1R is essential in infection, tissue trauma, or inflammatory alterations since it is not constitutively expressed as a B2R; instead, it is induced *in vivo* and *in vitro* by endotoxin, cytokines, and growth factors [9,32]. The use of knockout models, while informative, only partially mimics the clinical scenario where partial receptor activity might still be present, potentially limiting the applicability of the results to human conditions. However, our findings suggest new avenues for exploring KKS’s role in infection, tissue trauma, and inflammation.

Orthodontic force induces the expression of proinflammatory cytokines [34], and orthodontic appliances can significantly alter the oral microbiota, potentially increasing the number of periodontopathogenic species [37]. Furthermore, a possible carry-over effect may occur, where the treatment effect at one site may influence another site in split-mouth designs [38]. Consequently, this locally altered environment could incite B1R expression in the entire maxillary alveolar bone. However, the increased expression of B1R may not fully account for the phenotype observed in B2R^−/−^ mice.

In conclusion, this study underscores the critical roles of kinin receptors in regulating alveolar bone remodeling and osteoclastogenesis under both physiological and mechanically induced conditions. B2R deletion significantly increased osteoclastic activity, spontaneous ABL, and altered bone microarchitecture, emphasizing the receptor’s importance in maintaining bone homeostasis. The compensatory upregulation of B1R in the absence of B2R suggests functional redundancy; however, the observed phenotype in B2R^−/−^ mice cannot be solely attributed to this compensatory mechanism. The interplay between inflammatory mediators, particularly within the RANK/RANKL/OPG system, along with the differential expression of kinin receptors, further elucidates the complex regulatory network governing bone remodeling. These insights highlight the profound and intricate complexity of the KKS pathway in bone physiology and contribute to a deeper understanding of the molecular mechanisms involved in bone pathophysiology. This understanding has the potential to significantly impact future therapeutic strategies for managing bone-related disorders, offering hope for improved treatments. However, further research is warranted to explore the specific pathways and interactions that mediate these effects, focusing on the role of bone biomodulators.

## Supporting information

S1 Checklist ARRIVE 2.0 guidelines checklist. (PDF)

S2 Dataset Research results dataset.
(XLSX)
